# Access to and equitable distribution of COVID-19 vaccine in low-income countries

**DOI:** 10.1038/s41541-021-00323-6

**Published:** 2021-04-14

**Authors:** Krishna Prasad Acharya, Tirth Raj Ghimire, Supram Hosuru Subramanya

**Affiliations:** 1 Animal Quarantine Office, Budhanilkantha, Kathmandu, Nepal; 2 Animal Research Laboratory, Faculty of Science, Nepal Academy of Science and Technology (NAST), Khumaltar, Lalitpur, Nepal; 3 Manipal College of Medical Sciences, Pokhara, Nepal

**Keywords:** Immunology, Adaptive immunity, Epidemiology

The ongoing Coronavirus Disease 2019 (COVID-19) pandemic caused by SARS-CoV-2 still poses significant health challenges globally. The Harvard group’s models predict that a resurgence of SARS-CoV-2 could occur as late as 2024 after a period of apparent elimination, if the duration of immunity is intermediate and if other corona viruses induce intermediate cross-immunity^1^. Among more than 60 vaccine candidates in clinical trials, currently, only the Pfizer-BioNTech, Moderna COVID-19, and Johnson & Johnson COVID-19 vaccines have received Emergency Use Authorization (EUA) for active immunization to prevent COVID-19 from the US Food and Drug Administration (FDA)^2–4^. The Oxford-AstraZeneca COVID-19 vaccine has additionally received approval in the European countries, India, Argentina, Mexico, Brazil, Pakistan, Nepal, and others^5^. For emergency use, other vaccines like Sputnik V, BBIBP-CorV, CoronaVac, Ad5-nCoV, EpiVacCorona, and BBV152 have been approved in many countries in the world^6^. The approval of the vaccines has created some degree of confidence, and many countries have begun administering them. However, the accessibility of the vaccines to low-income countries (LICs) may be hindered, and thus, many concerned authorities have been questioned. It is because most of the vaccines have been reported to be reserved by wealthy nations^6,7^. These issues are extensively reviewed by the Lancet Commission on COVID-19 Vaccines and Therapeutics Task Force Members^8^.

As of February 19, 2021, about 90 countries had access to at least one COVID-19 vaccine. Gibraltar and Israel had more than 78 cumulative COVID-19 vaccination doses administered per 100 people in this context. Compared to this, Cambodia, Pakistan, Mauritius, Albania, Ecuador, Guyana, and Bolivia had less than 0.1 doses administered^6^. Until this period, ten countries that account for 60% of the global gross domestic product had administered 75% of all COVID-19 vaccines^9^. Dr. Tedros Adhanom Ghebreyesus, the WHO Director-General, and Henrietta Fore, UNICEF Executive Director, have pointed out that there are 130 countries, with a total population of 2.5 billion, that are yet to administer a single dose^10^. It is a pity that expert health workers are dying in sub-Saharan Africa^9^, indicating an international moral failure in these regions although six hundred thousand doses of the AstraZeneca-Oxford vaccine, produced by the Serum Institute of India have recently reached in Ghana. In summary, most countries in Africa and a few in Asia and South America are in the risk groups for vaccine inaccessibility. Antonio Guterres, the Chief of the United Nations (UN), has stated that progress on COVID-19 vaccinations has been wildly uneven and unfair (https://twitter.com/antonioguterres). Scientists believe that this uneven pattern of inoculations could also lead to virus mutations and new vaccine-resistant variants.

The COVID-19 pandemic has resulted in the deaths and severe illness of many people and the disruption of normal lives, a loss of jobs, a loss of trade, and has dwindled the already weak national economies in the LICs. In this situation, equitable access to a suitable and effective vaccine, especially for the front-line workers, is critical for mitigating and maintaining public health systems and economic growth. That is why the demand for COVID-19 vaccines has been soaring, although supply has been limited. Many underlying causes of vaccine inequity exist in the LICs, which we now discuss in turn.

First, many LICs have low socio-economic status with low levels of education, income, and occupation. These factors may directly affect the vaccine-purchasing and accepting processes of their people. Second, the geographical landscape of many LICs poses a significant challenge to vaccine distribution. Many high altitudinal landscapes within Hindu-Kush Himalayan regions, such as Nepal, Bhutan, Pakistan, and Afghanistan, make it very difficult for the vaccine campaigners and staff to distribute vaccines. The difficult situation might be worsened in the desert and remote areas engulfed in war, instability, and conflict. In this context, more than 160 million people have been estimated to be at risk of inaccessibility of the COVID-19 vaccine in Yemen, Syria, South Sudan, and Ethiopia^11^.

Third, people from urban slums and marginalized and migratory populations have poor access to immunization facilities. Vaccine distribution is challenging in urban and peri-urban slums that are overgrowing in developing countries. Fourth, most of the available COVID-19 vaccines need to be transported and stored at refrigerating to freezing temperatures, for example, the Oxford-AstraZeneca COVID-19 vaccine at 2–8 °C and the Pfizer vaccine at −70 °C, although new stability data submitted by the companies to the US regulator show that the latter vaccine can be stored at temperatures of −15 to −25 °C for up to 2 weeks^12^. Even to protect their quality, care is still needed after transferring these vaccines to the refrigerator or following thawing. Strict regulations for temperature are critical for the maintenance of efficacy, potency, and stability of vaccines. These are significant challenges in LICs due to a shortage of cold chain infrastructures and a lack of advanced technology to monitor the cold chain for storage, distribution, and transportation of vaccines, especially in the rural regions^13–15^. It could result in low immunization coverage in these areas and, subsequently, the probable endemicity of COVID-19 infections.

Fifth, levels of vaccine hesitancy, fear, and confusion have been raised in many countries because of the range of data from efficacy trials for the same product. For example, the Sinovac, a Chinese company, showed 50–91% efficacy^16,17^. Also, there is the apparent doubt whether the vaccines that have been designed and developed by the researchers following one year of the experiment will work against new variants of the virus. In this context, it is not easy for a developing nation to decide to spend a considerable amount of money to purchase the old vaccines or wait for other future products that would work against new variants.

Finally, obtaining intellectual property (IP) of COVID-19 vaccines by the developing countries from vaccine developers has not been entirely successful yet^18^. Although competitions may exist between many pharmaceutical companies, they are welcoming any interested company and country to license their intellectual property for COVID-19 vaccines. It has assured that vaccines should be global public innovations. Based on this principle, Moderna announced that it allows open access to the relevant IP for the pandemic period and is planning to out-license the IP during the post-pandemic period^19^. Notably, unrestricted access to IP of COVID-19 vaccine has been a boon for few countries like India, the Republic of Korea, Brazil, Indonesia, and South Africa, which have already started producing vaccines. However, LICs cannot easily take advantage of this due to their lack of domestic vaccine manufacturing capacity and thus rely on rich countries or their vaccine-developing companies. Most LICs lack vaccine-producing resources like policy, planning, programs, vaccinologists, organized laboratories, industries, Research & Developments, and government funding. Thus, even if a license is given to these countries, it will not solve vaccine production problems.

Currently, rich countries have been blamed for the underlying unequal distribution of COVID-19 vaccines around the globe. That may not be entirely true. These countries have contributed their efforts in resources and funding, and partnership with other countries. For example, the activities of the US toward the COVID-19 Vaccine Global Access (COVAX) facility, co-led by the Global Alliance for Vaccines and Immunisation (GAVI), the Coalition for Epidemic Preparedness Innovations (CEPI), and WHO are crucial. Although Trump’s government withdrew from WHO in July 2020, accusing it of being a puppet of China during the COVID-19 pandemic, the Biden Government is currently planning to join COVAX and rejoin the WHO. COVAX is working for quick, fair, safe, and global equitable access to COVID-19 vaccines, and thus, it is one of the best options for LICs to combat the pandemic. COVAX aims to vaccinate at least 20% of the people in 92 LICs of Africa, Asia, and Latin America by the end of 2021 as a first step^20^. Although affluent COVAX participants are criticized for stockpiling vaccines for themselves, COVAX might be proved a boon for the LICs if such confusion and criticism are removed.

Lessons on how a developed country can facilitate vaccine equity can be learned from the example of the UK. The UK ideally and practically has been ready to work for equitable global access to the COVID-19 vaccine^11^ by negotiating for temporary cease-fires in affected countries and facilitating distributing the vaccines for the people in those warzones. In a speech to a virtual G7 meeting, Borris Johnson, the UK Prime Minister, pledged to provide most of the excess doses of vaccine in the UK to distribute to poor countries^21^, which is a praiseworthy initiation by the world leader in fight against COVID-19 pandemic. In conclusion, the availability of the vaccine for LICs under the COVAX program^22^ and government efforts will play an essential role in achieving global immunity to stop the transmission of the virus. But the LICs should also receive support from high-income countries, private partners, UNICEF, GAVI, WHO, and other global humane organizations to have equitable access to the COVID-19, so there can be vaccines for all.
